# FLT-PET for the assessment of systemic sarcoidosis including cardiac and CNS involvement: a prospective study with comparison to FDG-PET

**DOI:** 10.1186/s13550-020-00742-x

**Published:** 2020-12-10

**Authors:** Patrick Martineau, Matthieu Pelletier-Galarneau, Daniel Juneau, Eugene Leung, Pablo Nery, Rob deKemp, Rob Beanlands, David Birnie

**Affiliations:** 1 Functional Imaging Department, BC Cancer Agency, 600 W 10th Ave, Vancouver, BC V5Z 4E6 Canada; 2 Gordon Center for Medical Imaging, Massachusetts General Hospital, Harvard Medical School, Boston, MA USA; 3 Department of Medical Imaging, Montreal Heart Institute, Université de Montréal, Montreal, QC Canada; 4 Centre Hospitalier de L’Université de Montréal (CHUM), Université de Montréal, Montreal, QC Canada; 5 Molecular Function and Imaging Program, The National Cardiac PET Centre and the Arrhythmia Service, Division of Cardiology, Department of Medicine, University of Ottawa Heart Institute, Ottawa, Canada; 6 Division of Nuclear Medicine, Department of Medicine, The Ottawa Hospital, Ottawa, ON Canada; 7 Arrhythmia Service, Division of Cardiology, Department of Medicine, University of Ottawa Heart Institute, 40 Ruskin Street – Room H 1285, Ottawa, ON K1Y 4W7 Canada

**Keywords:** Sarcoidosis, Neurosarcoidosis, Cardiac sarcoidosis, Fluorothymidine, FLT-PET, FDG-PET

## Abstract

**Background:**

2-deoxy-2-[18F]fluoro-d-glucose’s (FDG) biodistribution limits the evaluation of cardiac sarcoidosis (CS) and neurosarcoidosis (NS). While protocols for cardiac suppression exist, they can be inconvenient for patients and lead to incomplete cardiac suppression in many cases. Furthermore, FDG PET is limited in the detection of neurosarcoidosis due to an inability to suppress high level of physiological uptake within the brain. 3′-deoxy-3′-[^18^F]fluorothymidine (FLT) has been shown to accumulate in sarcoidosis lesions and this tracer lacks significant physiological myocardial and brain uptake, suggesting that this tracer may be useful for the assessment of sarcoidosis, including CS and NS, without the need for patient preparation. This prospective pilot study examined the performance of FLT vs FDG PET for systemic sarcoidosis, including cardiac and neural involvement.

**Materials and methods:**

Fourteen subjects with sarcoidosis were prospectively recruited and imaged with FDG- and FLT-PET. Two blinded, experienced readers independently reviewed the FLT-PET and FDG-PET images. Lesion distribution was compared between FLT and FDG. Agreement between FLT- and FDG-PET was determined using Cohen’s kappa and the intra-class correlation coefficient. Inter-observer variability of FLT and FDG-PET was assessed.

**Results:**

Twelve subjects had CS as per Heart Rhythm Society criteria and 1 had NS. FLT-PET was positive in 12 (86%), and FDG-PET in 11 (79%), with cardiac uptake present in 6 (50%) and 7 (58%) of subjects with CS, respectively. The subject with NS demonstrated uptake on both FLT and FDG-PET, with more lesions on FLT. There were no significant differences in the anatomical distribution of lesions between FLT and FDG. SUVs were significantly (*p* < 0.001) higher for FDG than FLT (5.8 ± 3.0 vs 2.3 ± 1.1, respectively), but not (*p* = 0.90) after adjusting for blood pool activity (2.8 ± 1.4 vs 2.8 ± 1.1, respectively). Agreement between FLT- and FDG-PET was good to excellent for the diagnosis of sarcoidosis, lung involvement, CS, and NS (*κ* = 0.76, 0.69, 0.86, and 1.0, respectively). Inter-observer agreement for FLT was excellent for diagnosing sarcoidosis, CS and NS (*κ* = 0.81, 0.85, and 1.0, respectively) and comparable to that of FDG.

**Conclusions:**

FLT-PET may be useful for the assessment of systemic sarcoidosis, as well as cardiac and neural involvement.

## Introduction

The last few years have seen an increased recognition of the importance in the detection of cardiac sarcoidosis (CS), accounting for up to 85% [1] of sarcoid deaths. In addition, neurosarcoidosis (NS) is associated with a 10% mortality rate [2] and present in 5–15% of cases [3]. For both NS and CS, post-mortem studies have revealed that only a fraction of cases are diagnosed antemortem with up to 39% and 50% of cases of CS and NS detected on autopsy [4].

2-deoxy-2-[^18^F]fluoro-d-glucose (FDG)-PET is useful in diagnosing and assessing the extent of active sarcoidosis, due to its ability to image inflammation. FDG-PET has been shown to be useful in identifying appropriate biopsy sites in patients with sarcoidosis as affected lymph nodes are often morphologically unremarkable and undetectable by other imaging modalities [5]. Furthermore, the degree of FDG uptake, particularly within lung parenchyma, has been shown to serve as an effective therapeutic target [6] with the degree of lung uptake decreasing following the initiation of therapy [7]; however, despite a near perfect sensitivity of 96–100% for pulmonary disease [8], the diagnostic accuracy for cardiac involvement is lower, with one meta-analysis reporting a sensitivity/specificity of 89%/78% [9]. Furthermore, extensive patient preparation is required to suppress physiological FDG uptake and non-diagnostic or false positive studies due to suboptimal suppression can be seen in 10–20% of cases [10]. The role of FDG-PET in assessing NS remains largely unstudied.

3′-deoxy-3′-[^18^F]fluorothymidine (FLT)-PET has recently been shown to be useful in the assessment of CS without requiring extensive pre-imaging preparation [11, 12]. This prospective study investigated the use of FLT-PET for the evaluation of sarcoidosis, including myocardial and neural involvement, with comparison to FDG-PET.

## Methods

### Subjects

Subjects with tissue-proven sarcoidosis scheduled for a whole-body FDG-PET, perfusion PET and dedicated cardiac FDG-PET for the assessment of CS were considered for enrollment. Exclusion criteria included breastfeeding, pregnancy, claustrophobia, inability to lie still in a supine position, unwillingness or inability to provide informed consent, age < 18 years, or active malignancy. The results of other relevant imaging studies, histopathology findings, and the subject’s clinical histories were collected. All participants were subsequently imaged with FLT-PET (whole-body and ECG-gated cardiac images) within 2 weeks of FDG-PET imaging. Subject treatment was neither initiated nor changed in the interval between FDG and FLT imaging. This prospective single-center study was approved by the institutional research ethics board and all participants provided signed informed consent.

## Subject preparation and imaging protocol

Imaging studies were performed in the following order: (1) cardiac rest perfusion scan; (2) whole-body FDG scan; (3) ECG-gated cardiac FDG scan. FLT studies were acquired in the following order: (1) whole-body FLT scan; (2) ECG-gated cardiac FLT scan. All images were acquired in 3D mode and reconstructed using an iterative technique. PET images were performed on a Discovery 690 or 600 PET/CT scanner (GE Healthcare, Waukesha, WI). FLT scans were performed within 14 days of perfusion and FDG scans.

### Perfusion PET

Following administration of either ^82^Rb (10 MBq/kg) or [^13^N]NH_3_ (3–5 MBq/kg), subjects underwent rest ECG-gated perfusion PET, with a low-dose CT for localization and attenuation correction. Rest perfusion images were reviewed using the standard 17-segment model [13] and a 5-point grading system (0 = normal radiotracer uptake, 1 = mildly reduced, 2 = moderately reduced, 3 = severely reduced, 4 = absence of uptake).

### FDG-PET

Subjects were instructed to follow a low-carbohydrate, high fat, protein-permitted diet (subjects were provided with detailed dietary instructions) followed by a fast for at least 12 h. Low-dose unfractionated heparin was intravenously injected prior to FDG administration in participants without contraindication [10]; 5 MBq/kg of FDG was injected intravenously followed by an uptake phase of 60 min and whole-body imaging. A low-dose, non-contrast CT scan from the femoral region to the head was performed for attenuation correction; 90 min after FDG injection, a 20-min ECG-gated cardiac acquisition was performed.

### FLT-PET

For FLT-PET imaging, participants were instructed to not eat or drink anything except water for six hours prior to imaging. No other preparation was specified.

Intravenous injection of 370 MBq of FLT was followed by an uptake phase of 60 min. The FLT-PET imaging was otherwise performed in an identical fashion to the FDG-PET acquisition.

## Image interpretation

Images were anonymized and reviewed in random order by two experienced nuclear medicine physicians (PM and DJ) blinded to any identifying information using HybridViewer PET-CT fusion software (Hermes Medical Solutions). CT findings were reviewed and used for anatomic localization and characterization of PET findings. Perfusion images and dedicated cardiac DG-PET and FLT-PET were reviewing using 4DM (INVIA Medical Imaging Solutions). Regions of interest (ROIs) were defined as spheres 1 cm in diameter. All reported SUVs consisted of SUV_Max_ measured within an ROI. Blood pool SUVs were measured in a similar fashion using an ROI within the ascending thoracic aorta. Individual lesions were defined as foci of radiotracer uptake significantly greater than background and visually distinct from the normal biodistribution of the radiotracer. Active sarcoidosis was defined as uptake of either FDG or FLT in lesions consistent with sarcoidosis. A third reader resolved differences in interpretation.

## Reference standard

The gold standard for sarcoidosis was tissue diagnosis while the gold standard for cardiac involvement consisted of the Heart Rhythm Society criteria [14]. Clinical diagnosis was considered the gold standard for NS.

## Statistical analysis

Descriptive statistics are presented as the number (percent) for categorical variables and mean (± standard deviation) for continuous variables. Normality was tested using the Shapiro–Wilk test. Differences between groups were compared using Student’s paired samples *t* test. Kappa (*κ*) statistics were calculated for inter-observer agreement of categorical findings on FLT- and FDG-PET scans while continuous variables were compared with the intra-class correlation coefficient (ICC) using a two-way random effects, absolute agreement, single rater model [15]. Interpretation of concordance measures was in keeping with the guidelines of Cicchetti et al*.* [16]. A *p* value of < 0.05 was considered statistically significant. All analyses were performed using SPSS v20 (IBM Corp. Released 2011. IBM SPSS Statistics for Windows, Version 20.0. Armonk, NY: IBM Corp).

## RESULTS

### Baseline demographics

Fourteen subjects were recruited between January 10^th^, 2017 and November 15^th^, 2017. Subject characteristics are shown in Table 1. Extra-cardiac sarcoidosis was confirmed in all subjects by lymph node tissue sampling. 12 (86%) were diagnosed with CS, in accordance with the Heart Rhythm Society (HRS) criteria [14]. All subjects positive for CS had a histological diagnosis of extra-cardiac sarcoidosis, in addition to an appropriate cardiac presentation and/or cardiac MRI findings compatible with CS. Subjects with CS were diagnosed independently of the PET findings in this study (Table 1). 1 (7%) subject had previously diagnosed NS.

**Table 1:** Subject baseline characteristics

Subject #	Age (yrs)	Gender	Ethnicity	Positive for CS by HRS criteria?	Cardiac presentation	Biopsy proven extra-cardiac sarcoidosis?	Initial assessment or reassessment?	On active treatment at the time of current imaging?	MRI findings compatible with CS?
1	44	Male	Caucasian	Yes	Bifascicular block	Yes	Reassessment	Yes, methotrexate/prednisone	Yes
2	64	Male	Caucasian	No	Cardiomyopathy	Yes	Initial	No	No
3	49	Male	Caucasian	Yes	Complete AV block	Yes	Initial	No	Yes
4	49	Female	African-American	Yes	Complete AV block	Yes	Initial	No	No
5	57	Male	Caucasian	Yes	Complete AV block	Yes	Reassessment	No	Not performed
6	56	Male	Caucasian	Yes	Cardiomyopathy	Yes	Reassessment	No	Not performed
7	54	Female	Caucasian	No	None	Yes	Initial	No	No
8	66	Male	Caucasian	Yes	Complete AV block	Yes	Reassessment	Yes, methotrexate	Yes
9	68	Male	Caucasian	Yes	None	Yes	Initial	No	Yes
10	57	Male	Caucasian	Yes	Ventricular tachycardia	Yes	Reassessment	Yes, prednisone	Yes
11	54	Female	Caucasian	Yes	Complete AV block	Yes	Initial	No	No
12	57	Male	Caucasian	Yes	Complete AV block	Yes	Initial	No	Yes
13	58	Female	Asian	Yes	Complete AV block	Yes	Initial	No	Yes
14	55	Female	Asian	Yes	Complete AV block	Yes	Initial	No	Not performed

## Imaging

### Whole-body findings

Image quality for all PET studies was excellent. Of the 14 subjects, 12 (86%) showed FLT and 11 (79%) FDG uptake compatible with active sarcoidosis. A single subject (#8) demonstrated mild uptake (SUV 1.6 compared to a blood pool SUV of 1.4) in a few mediastinal lymph nodes on FLT, with no other uptake and without uptake on the corresponding FDG study. This subject was receiving treatment with methotrexate at the time of imaging. The distribution of lesions is shown in Table 2 with the most commonly affected sites consisting of thoracic lymph nodes (71% of FLT and FDG cases) and cervical lymph nodes (50% of FLT and 57% of FDG cases). Comparison of the anatomical distribution of lesions revealed no statistically significant differences (Table 2).

**Table 2:** Anatomical distribution of sarcoidosis lesions on FLT- and FDG-PET (*N* = 14)

Anatomical site	FLT-PET	FDG-PET	*p* value	Agreement^†^
*Lymph nodes*				
Thoracic	10/14 (71%)	10/14 (71%)	0.99	0.65 (95% CI 0.21 to 1.0)
Cervical	7/14 (50%)	8/14 (57%)	0.72	0.29 (95% CI − 0.21 to 0.78)
Abdominal/pelvic	1/14 (7.1%)	5/14 (36%)	0.077	0.32 (95% CI − 0.18 to 0.81)
*Heart*				
Left ventricle	6/14 (43%)	7/14 (50%)	0.72	0.86 (95% CI 0.59 to 1.0)
Right ventricle	2/14 (14%)	4/14 (29%)	0.38	0.59 (95% CI 0.11 to 1.0)
*Central nervous system*				
Intracranial	1/14 (7.1%)	0/14 (7.1%)	0.33	0.0 (95% CI 0.0 to 0.0)
Spinal/paraspinal	1/14 (7.1%)	1/14 (7.1%)	0.99	1.0 (95% CI 1.0 to 1.0)
*Lung parenchyma*				
–	5/14 (36%)	5/14 (36%)	0.99	0.69 (95% CI 0.29 to 1.0)
*Salivary glands*				
–	0/14 (0%)	1/14 (7.1%)	0.33	− 0.11 (95% CI − 0.26 to 0.04)
*Spleen*				
–	0/14 (0%)	1/14 (7.1%)	0.33	0.0 (–)
*Liver*				
–	0/14 (0%)	0/14 (0%)	–	–
*Bone*				
–	0/14 (0%)	0/14 (0%)	–	–
*Average number of sites/per subject with active disease*				
–	1.6 (1.0)	1.8 (1.3)	0.75	0.78 (95% 0.45 to 0.93)

FLT-PET = 3′-deoxy-3′-[^18^F]fluorothymidine positron emission tomography, FDG-PET = 2-deoxy-2-[18F]fluoro-d-glucose positron emission tomography

^†^Agreement between categorical and continuous variables was assessed using Cohen’s kappa and the intraclass correlation coefficient, respectively

**Fig. 1:**
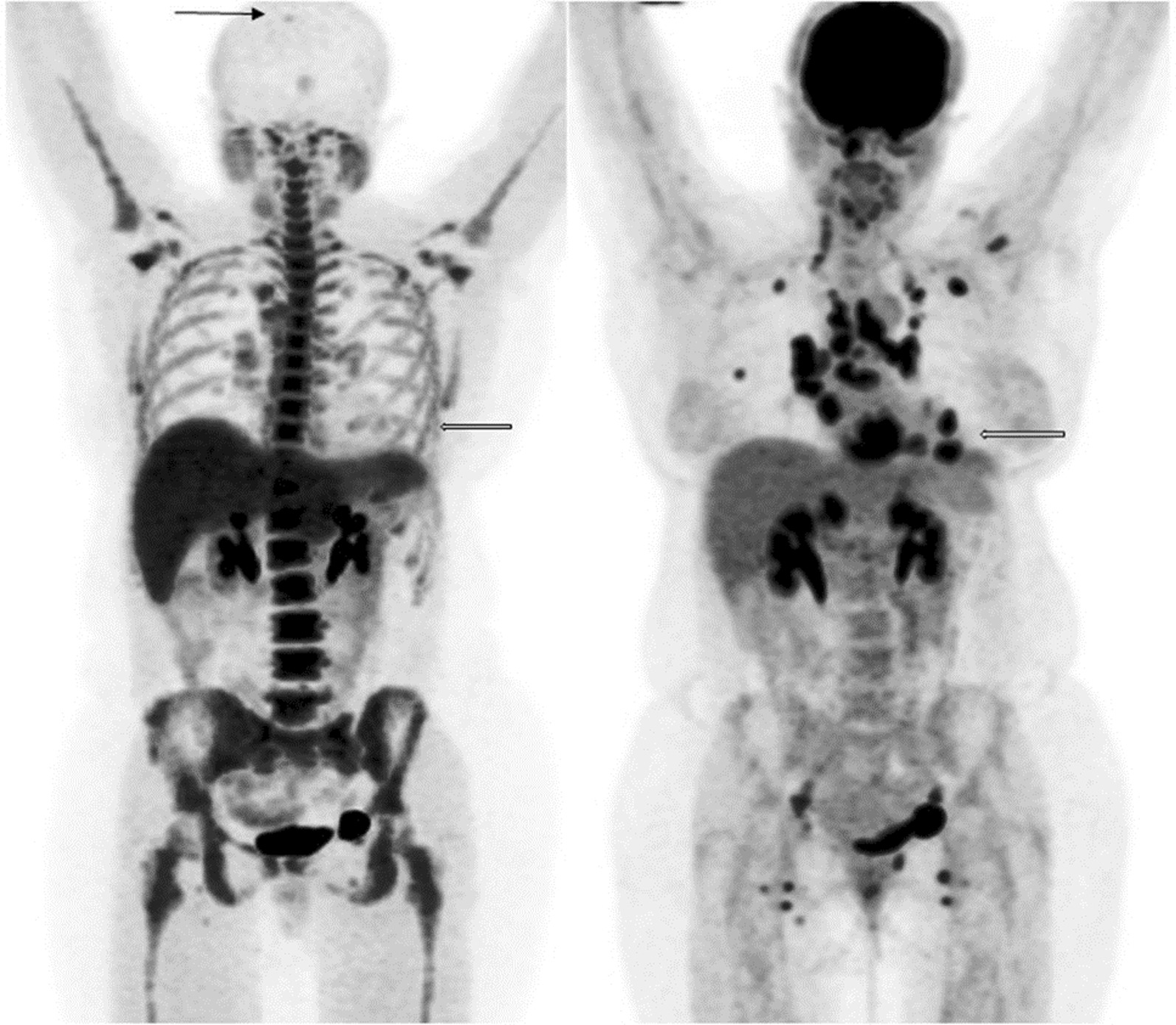
Whole-body maximum intensity projections of FLT-PET (left) and FDG-PET (right) studies in the same subject (subject #4) with evidence of sarcoidosis, including cardiac and neural involvement (arrows)

Figure 1 shows an example of whole-body FLT and FDG studies in the same subject with sarcoidosis and evidence of active CS and NS. Agreement between FLT- and FDG-PET for the assessment of individual sites varied but was highest for cardiac [*κ* = 0.86 (95% CI 0.59–1.0)] and CNS (spinal/paraspinal) [*κ* = 1.0 (95% CI 1.0–1.0)] involvement (Table 2). Agreement for the involvement of lung parenchyma was good [*κ* = 0.69 (95% CI 0.29–1.0)] while the agreement for the total number of affected sites was excellent [ICC = 0.78 (95% 0.45–0.93)]. Overall, agreement between FLT- and FDG-PET for the diagnosis of active sarcoidosis was excellent (*κ* = 0.76 (95% CI 0.32–1.0), Table 3). Inter-observer agreement for active sarcoidosis was excellent for both FLT and FDG (*κ* = 0.81 (95% CI 0.46–1.0) vs *κ* = 1.0 (95% CI 1.0–1.0, respectively), Additional file 1: Table S1).

**Table 3:** Comparison of FLT- and FDG-PET for the diagnosis of active sarcoidosis [*κ* = 0.76 (95% CI 0.32–1.0)]

	FLT-PET		Total
	Positive scan consistent with active sarcoidosis	Negative scan	
*FDG-PET*			
Positive scan consistent with active sarcoidosis	11	0	11
Negative scan	1	2	3
Total	12	2	14

**Fig. 2:**
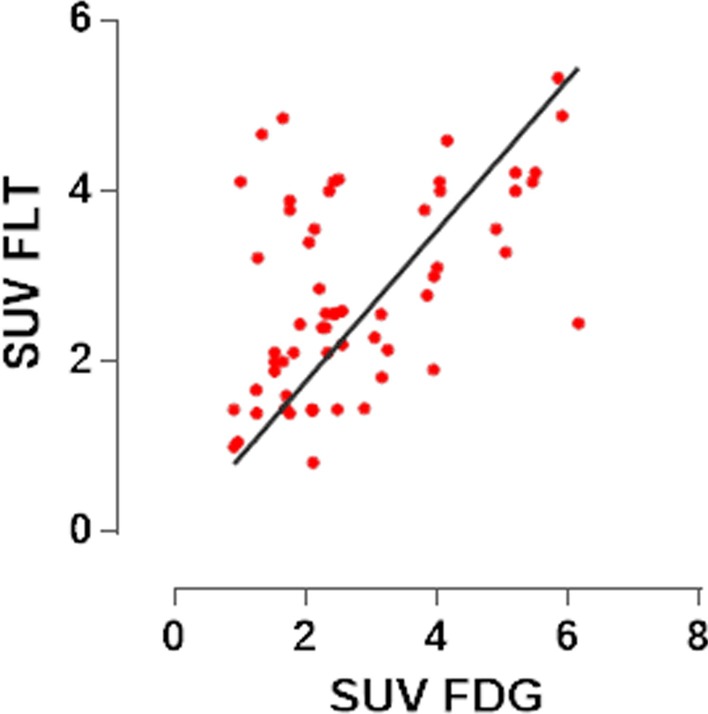
Lesion-per-lesion comparison of blood-pool corrected FLT and FDG SUVs. The linear regression equation was *Y* = 0.89*X

Mean SUV of sarcoidosis lesions differed significantly (*p* < 0.001) between FLT and FDG (2.3 ± 1.1 and 5.8 ± 3.0, respectively); however, the difference was no longer significant (*p* = 0.90) after adjusting for blood pool activity (FLT, FDG: 2.8 ± 1.1, 2.8 ± 1.4). Blood pool activity was significantly less (*p* < 0.0001) for FLT than FDG (1.2 ± 0.2, 2.4 ± 0.4, respectively). Linear regression was used to compare the blood pool adjusted SUV for FLT and FDG on a lesion-per-lesion level, for a total of 61 paired lesions (Fig. 2). The resultant equation is shown in Fig. 2 and was associated with an R of 0.51.

### Cardiac PET findings

**Fig. 3:**
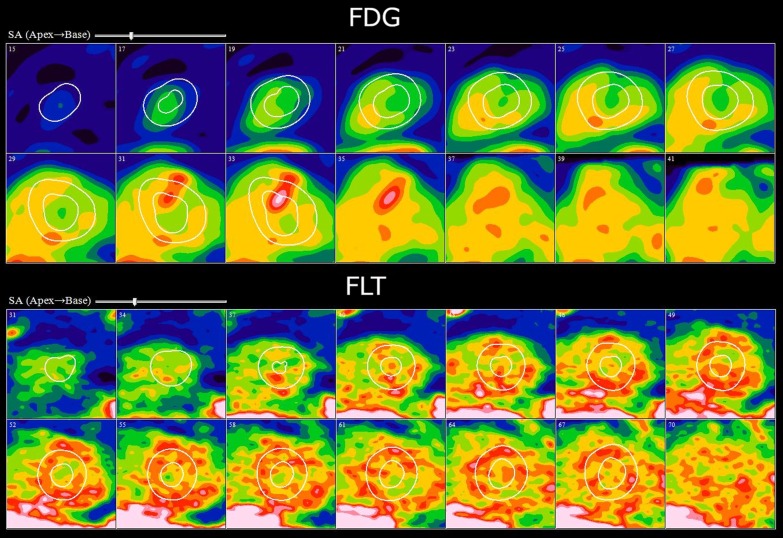
Cardiac short axis views of subject #10 demonstrating a focus of myocardial uptake in the basal anterior wall on FDG-PET (top) without corresponding uptake on the FLT-PET study (bottom). The diffuse heterogeneous uptake seen on the FLT study is in keeping with background blood pool uptake

Additional details of the cardiac findings have been published separately [12]. 12 (86%) subjects underwent perfusion PET with [^13^N]NH_3_ and 2 (14%) with ^82^Rb. All cardiac PET studies were considered diagnostic. Of the 12 subjects positive by HRS criteria, 7 (58%) showed myocardial FDG uptake consistent with active sarcoidosis, while myocardial FLT uptake was present in 6 (50%). A single subject (#10) demonstrated evidence of active CS on FDG without corresponding FLT activity (Fig. 3). In this subject, abnormal FDG uptake was limited to a single cardiac segment with an SUV of 3.5 (blood pool 2.6). This subject was receiving treatment with prednisone at the time of imaging and findings of extra-cardiac sarcoidosis were present on both the FLT and FDG studies.

**Fig. 4:**
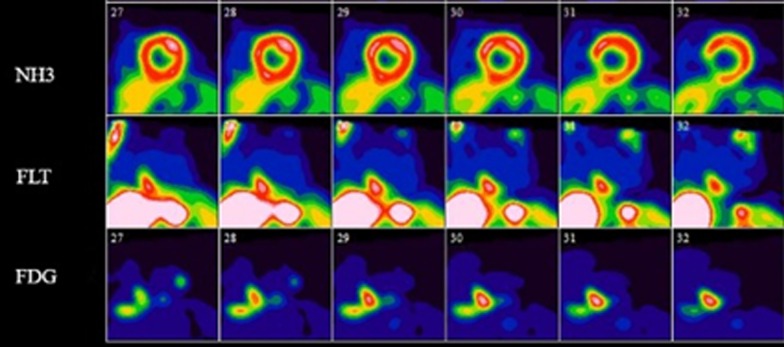
Selected short axis images of cardiac perfusion PET (top), FLT (middle), and FDG (bottom) studies in short, vertical, and horizontal long axes in a single subject with CS. The findings were consistent with active CS on both the FLT and FDG studies. The images show the relationship between an area of scarring in the inferior aspect of the ventricular septum which overlaps an area of concordant uptake on both FDG and FLT

**Fig. 5:**
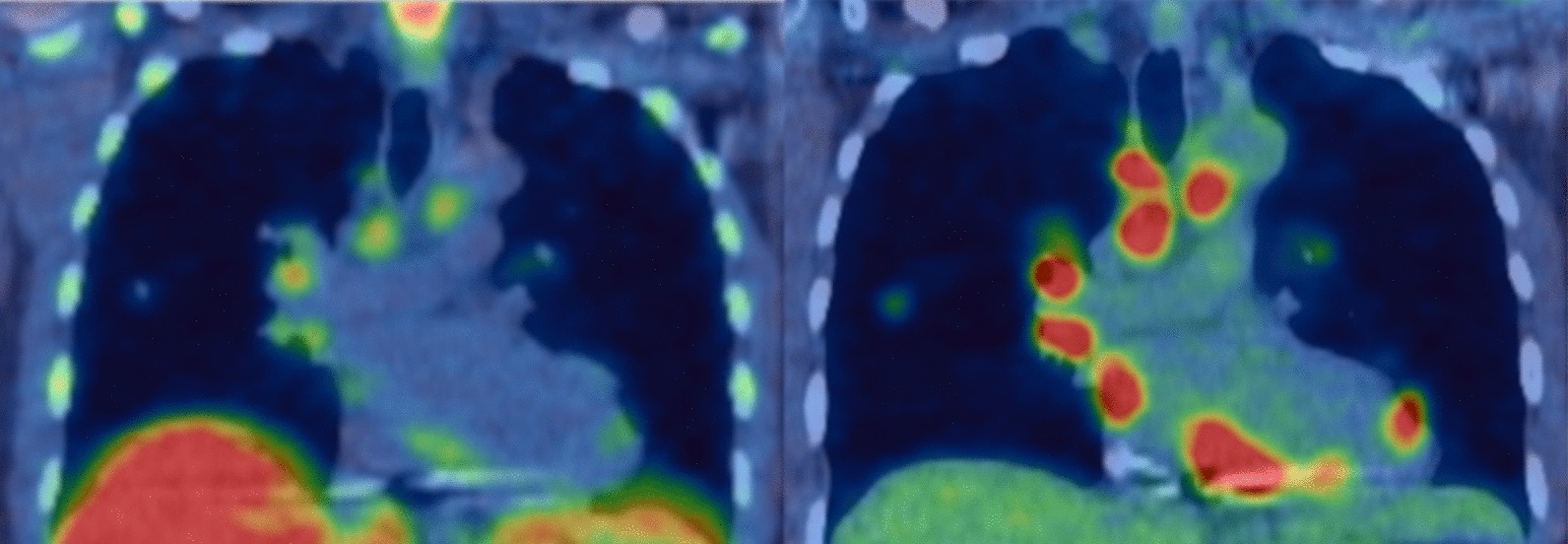
Coronal section through the mediastinum demonstrating thoracic lymphadenopathy and cardiac involvement in a single subject on FLT-PET (left) and FDG-PET (right)

**Fig. 6:**
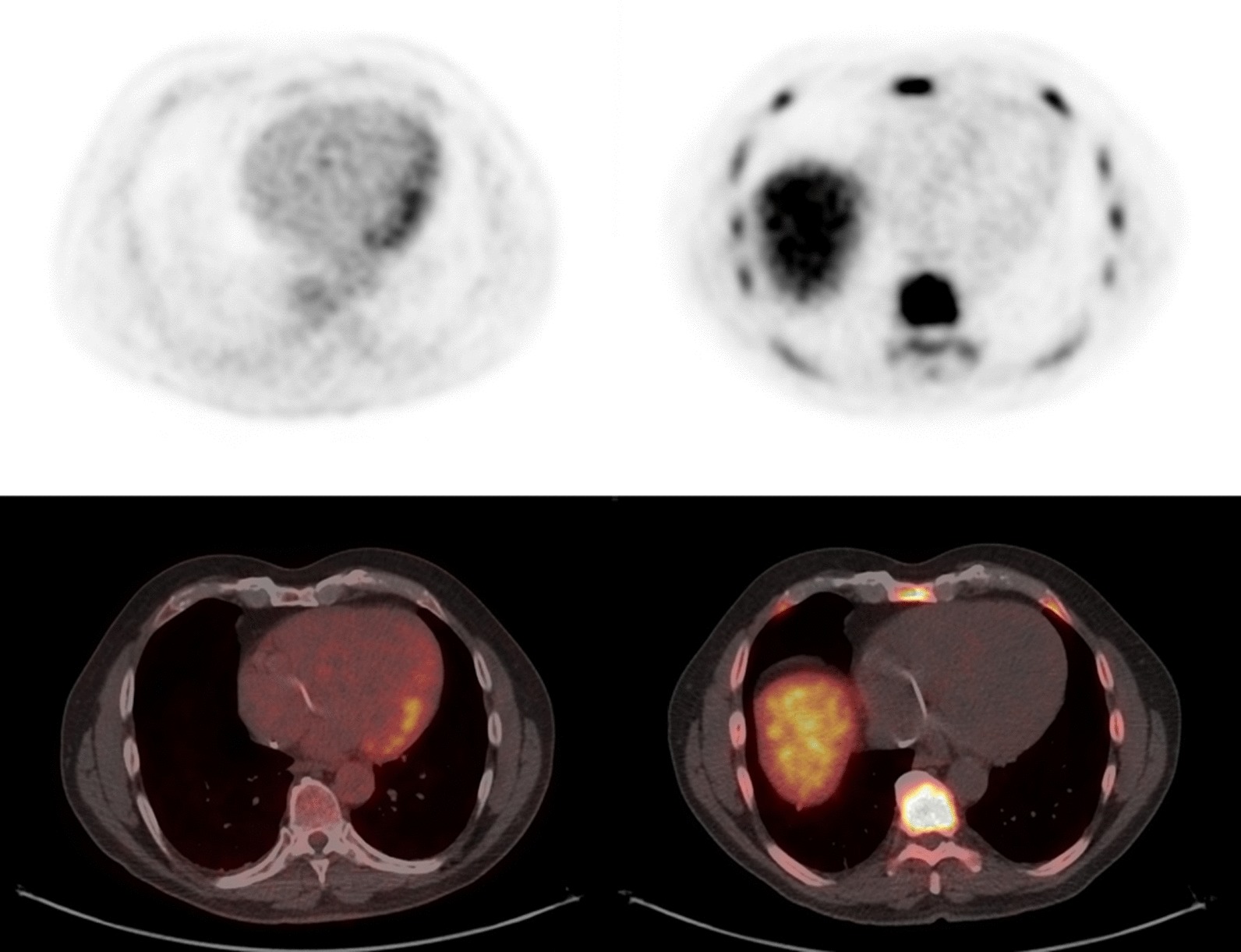
Subject demonstrating isolated diffuse lateral ventricular wall FDG uptake (left), a finding usually interpreted as a normal variant. No corresponding uptake was present on the FLT study (right)

An example of a perfusion PET, dedicated cardiac FDG-, and FLT-PET study in a subject with active CS is shown in Fig. 4 while Fig. 5 shows the relationship between cardiac findings and mediastinal uptake for FLT and FDG. All subjects with evidence of active CS had left ventricular (LV) uptake, while 4 and 2 of these had right ventricular (RV) uptake on FDG and FLT, respectively. None of our subjects had findings suggestive of isolated CS. One subject demonstrated isolated lateral wall myocardial FDG uptake (Fig. 6) without corresponding FLT uptake or perfusion deficits and was considered negative for active CS. The cardiac FDG findings in this subject were classified as negative. A summary of cardiac findings is shown in Table 4.

**Table 4:** Summary of cardiac PET findings in subjects with CS (*N* = 12*)

	FLT-PET	FDG-PET	*p* value
# of subjects with cardiac uptake (%)	6 (50%)	7 (58%)	0.70
# of LV segments involved ± SD	6.2 ± 1.5	7.3 ± 3.5	0.50
# of segments in LAD^†^ territory ± SD	2.5 ± 1.0	2.7 ± 1.3	0.75
# of segments in LCX territory ± SD	1.3 ± 1.0	1.7 ± 1.4	0.59
# of segments in RCA territory ± SD	2.5 ± 0.8	2.9 ± 1.5	0.61
# of subjects with RV uptake (%)	2 (17%)	4 (33%)	0.17
Maximum LV SUV ± SD	15.4 ± 4.4	13.7 ± 6.0	0.58
LV SUV_Total_ ± SD	27.7 ± 8.4	45.7 ± 33.1	0.22
Maximum RV SUV ± SD ^α^	2.4 ± 0.4	4.6 ± 5.0	0.59
	*Perfusion PET*		
# of subjects with perfusion defects (%)	10 (83%)		
SRS ± SD	6.2 ± 4.6		
SRS in LAD territory ± SD	3.2 ± 2.9		
SRS in LCX territory ± SD	1.0 ± 1.3		
SRS in RCA territory ± SD	2.0 ± 1.9		

*LV* left ventricle, *RV* right ventricle, *LAD* left anterior descending artery, *LCX* left circumflex artery, *RCA* right coronary artery, *SUV* standardized uptake value

*Only subjects satisfying the HRS criteria (*N* = 12/14) were included in this analysis

^†^Vascular territories defined as per AHA 17 segment model [13]

^α^Includes only those subjects with evidence of RV uptake

Concordance analysis of the cardiac findings in those subjects that satisfied the HRS criteria is shown in Table 5. Agreement between FLT and FDG for left and right ventricle involvement was excellent and fair, respectively, [left: *κ* = 0.86 (95% CI 0.59–1.0); right: *κ* = 0.59 (95% CI 0.082–1.0)]. When comparing the number of affected segments on FLT and FDG, the ICC was excellent overall [ICC = 0.81(95% CI 0.49–0.94)], as well as in the left anterior descending and right coronary artery territories (0.81 (95% CI 0.51–0.94) and 0.84 (95% CI 0.56–0.95), respectively). Agreement was fair [0.54 (95% CI 0.03–0.83)] in the left circumflex territory. Agreement between the various SUV measures on FLT and FDG was poor.

**Table 5:** Concordance analysis of cardiac findings between FLT and FDG-PET in subjects satisfying the HRS criteria for CS (*N* = 12)

	Agreement^†^
Subjects with LV uptake	0.81 (95% CI 0.47 to 1.0)
Subjects with RV uptake	0.57 (95% CI 0.08 to 1.0)
# of LV segments involved	0.81 (95% CI 0.49 to 0.94)
# of segments in LAD territory	0.81 (95% CI 0.51 to 0.94)
# of segments in LCX territory	0.54 (95% CI 0.03 to 0.83)
# of segments in RCA territory	0.84 (95% CI 0.56 to 0.95)
Maximum LV SUV	0.22 (95% CI − 0.38 to 0.69)
Maximum RV SUV	0.36 (95% CI − 0.24 to 0.76)
Mean segmental SUV	− 0.002 (95% CI − 0.58 to 0.57)
LV SUV_Total_	0.20 (95% CI − 0.42 to 0.70)

^†^Agreement between categorical and continuous variables was assessed using Cohen’s kappa and the intraclass correlation coefficient, respectively

Of the 12 subjects with CS, the combination of perfusion/FLT-PET and perfusion/FDG-PET was positive in 11/12 (92%) cases [*κ* = 1.0 (95% CI 1.0–1.0)]. Inter-observer agreement for active cardiac involvement was excellent for both FLT- and FDG-PET (FLT: *κ* = 0.85 (95% CI 0.57–1.0); FDG: *κ* = 0.72 (95% CI 0.38–1.0), Additional file 2: Table S2).

### Neurosarcoidosis findings

**Fig. 7:**
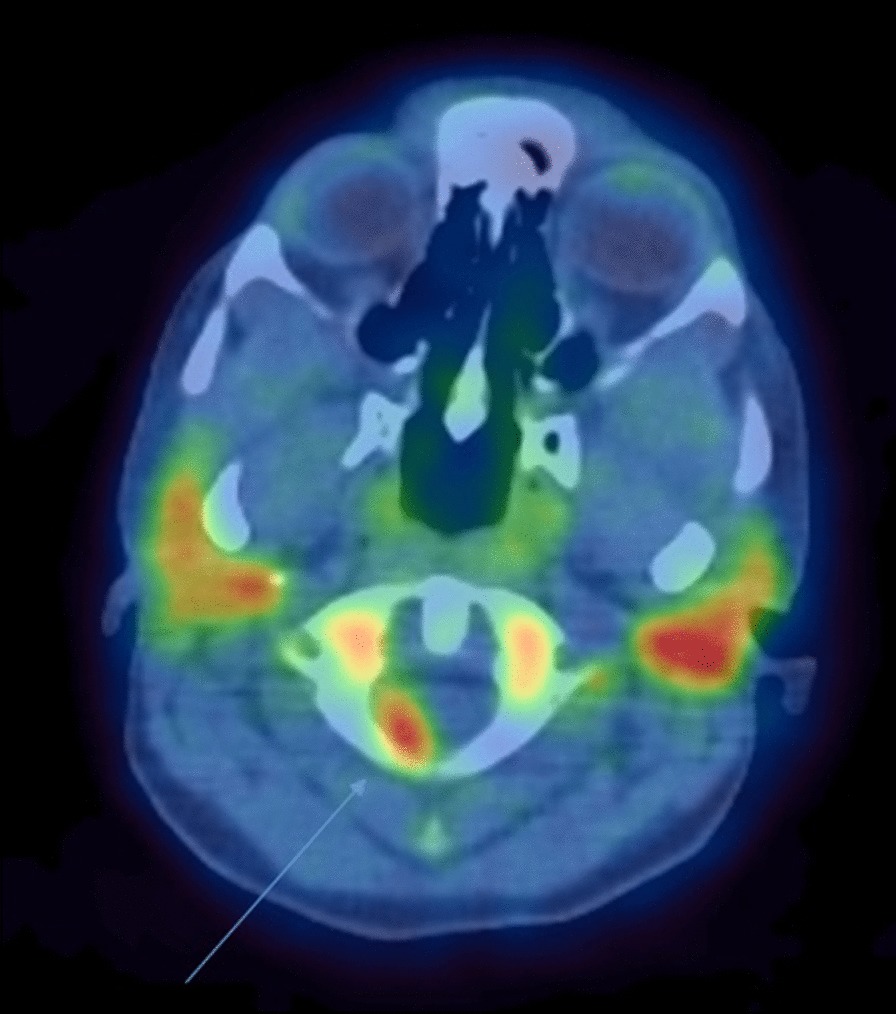
FLT-PET study in a subject with neurosarcoidosis (arrow) (subject #4)

A single case of NS was positive on both FLT and FDG (subject #4); however, FLT-PET was able to identify two paraspinal (Fig. 7) and one intracalvarial lesions (Fig. 1), while FDG-PET only detected the paraspinal lesions. The agreement between FLT-PET and FDG-PET for the diagnosis of active NS was excellent [*κ* = 1.0 (95% CI 1.0–1.0)] (Additional file 3: Table S3).

## Discussion

In this prospective study, FLT-PET demonstrated excellent imaging and diagnostic properties for the evaluation of extra-cardiac sarcoidosis, as well as CS. FLT-PET was able to detect multiple NS lesions in a single subject with known neural involvement.

Limitations inherent in cardiac FDG-PET imaging are well-known and include extensive patient preparation, variable success in suppression of myocardial activity, and the existence of normal patterns of myocardial uptake complicating the interpretation of studies [10]. In particular, interpretation of the various cardiac uptake patterns is associated with only moderate inter-observer agreement [17]. Due to high, non-suppressible physiological uptake seen in the central nervous system (CNS), FDG-PET plays very little role in the evaluation of NS, with the extant literature limited to case reports. Because of these limitations, there is a need for a radiotracer which can accurately assess not only the lung parenchyma and lymph nodes, but also the myocardium and CNS, without the need for extensive patient preparation.

FLT offers multiple advantages, especially for the assessment of CS and NS. In particular, lack of physiological myocardial uptake obviates the need for extensive patient preparation resulting in increased patient convenience, and decreased risk of non-diagnostic studies due to patient non-compliance with pre-imaging preparation instructions. Unlike FDG, there are no known normal variants demonstrating non-pathological myocardial FLT uptake, simplifying cardiac interpretation while lowering the risk of false-positive studies. In particular, our subject with a normal variant (i.e., lateral wall FDG uptake) had an unremarkable cardiac FLT study. In our subject with NS, both FLT and FDG were able to detect paraspinal lesions; however, only FLT adequately demonstrated intracalvarial disease, due to a lack of background cerebral uptake. The high concordance in the distribution of lesions seen on FLT and FDG at key sites (i.e., heart, CNS, lung parenchyma), as well as for the overall number of organs involved, suggests a comparable performance to FDG disease staging. Furthermore, FLT effectively identified small pathological lymph nodes which could be used to identify active sites of disease for diagnostic biopsy. FLT showed a non-significant trend towards lower sensitivity for the detection of abdominal lymph nodes compared to FDG—this may have be related to the observation that, in many patients, involved lymph nodes are commonly seen around the porta hepatis and the high hepatic uptake seen on FLT may make identification of lymph nodes at the porta challenging; however, further studies will needed to confirm this. Although not shown in our study, FLT-PET could, in theory, be less sensitive than FDG-PET for the detection of sarcoidosis lesions within the liver and bone marrow due to the significant uptake normally seen in these organs; however, this may deemed acceptable in light of the improved assessment of the heart and CNS and the relatively limited clinical impact of hepatic and marrow involvement.

Our study was not designed to determine the utility of FLT lung uptake as a therapeutic target; however, one would expect that, in order to duplicate the success of FDG in this respect, there should at least be good concordance between FLT and FDG for the assessment of parenchyma involvement, which was present in our results. Additional studies will be needed to assess the effectiveness of FLT-PET in the assessment of therapeutic response.

Although FLT demonstrates a lower degree of uptake (SUV) in sarcoidosis lesions when compared to FDG, the difference was not clinically significant with lesions demonstrating similar signal to background with both tracers. After correction for background activity (i.e., blood pool) the difference in SUVs was not statistically significant. Linear regression analysis revealed a relatively low degree of correlation between FDG and FLT uptake compatible with our previously reported results [12]. This can be attributed to the fact that the degree of FDG uptake is non-specific and reflects all underlying metabolic processes while FLT uptake is specific to proliferative activity.

FLT activity is expected to represent granuloma burden while FDG reflects general inflammatory activity. Differences in uptake mechanisms between FLT and FDG could be useful for disease monitoring and therapy assessment. In particular, we speculate that the specificity of the uptake mechanism of FLT may be particularly well-suited for monitoring response to therapy; however, additional studies are needed.

It is unclear why a single subject (#10) showed discordant myocardial findings (positive on FDG, negative on FLT) while another subject (#8) showed discordant whole-body FLT and FDG findings (positive on FLT, negative on FDG). In both cases, these subjects were undergoing treatment at the time of imaging. It may be that the discordance was related to treatment effects. Also, it should be noted that the imaging findings in both cases were subtle and the discordances might be attributable to limited sensitivity (i.e., false-negative) and/or specificity (i.e., false-positive) for either FLT or FDG.

Our results can be compared to those of Norikane et al*.* [11] who conducted a retrospective study involving 20 subjects with newly diagnosed cardiac sarcoidosis and compared FLT and FDG-PET findings. They found that both FLT and FDG had high accuracy for cardiac and extra-cardiac thoracic sarcoidosis. There are, however, several important differences between our studies. Our study was prospective and used the HRS criteria as gold standard, compared to the JMHW criteria used by Norikane et al. Furthermore, subjects undergoing cardiac evaluation in the Norikane study had a high rate of inconclusive FDG studies (4/20) while all of our subjects’ studies were diagnostic. Also, Norikane limited their imaging to the thorax while we acquired near whole-body images, which enabled us to compare findings in extra-thoracic sarcoidosis (including intracalvarial NS). Importantly, the work by Norikane et al*.* did not incorporate cardiac perfusion imaging in their analysis—we have previously shown significant associations between the findings on FLT-PET and perfusion imaging [12]. Finally, none of their subjects had evidence of NS.

Some limitations of this study should be acknowledged. Our sample size was small, and lack of negative controls prevented us from calculating the accuracy for whole-body sarcoidosis. In order to remedy this, a larger prospective study is underway. In addition, all subjects were referred for suspicion of CS. This subgroup of subjects may not be representative of typical subjects with sarcoidosis and may have influenced the distribution of sarcoidosis lesions we observed in our study. Finally, there was only a single subject with a clinical diagnosis of NS, limiting our assessment of the utility of FLT-PET for neural involvement.

In summary, FLT-PET has the ability to accurately detect sarcoidosis lesions, including cardiac and CNS involvement, without the need for extensive patient preparation. Follow-up studies are ongoing in order to confirm these findings.

## Supplementary information

**Additional file 1**. Table S1: Comparison of both readers' overall interpretation of the FLT- and FDG-PETs for active sarcoidosis [FLT *κ* = 0.81 (95% CI 0.46–1.0), FDG *κ* = 1.0 (95% CI 1.0–1.0)].

**Additional file 2**. Table S2: Comparison of both readers' overall interpretation of the FLT- and FDG-PETs for cardiac involvement [FLT *κ* = 0.85 (95% CI 0.57–1.0), FDG *κ* = 0.72 (95% CI 0.38–1.0)].

**Additional file 3**. Table S3: Comparison of both readers' overall interpretation of the FLT- and FDG-PETs for neural involvement [FLT *κ* = 1.0 (95% CI 1.0–1.0), FDG *κ* = 1.0 (95% CI 1.0–1.0)].

## Data Availability

The datasets used and/or analyzed during the current study are available from the corresponding author on reasonable request.

## Abbreviations

CNS: Central nervous system
CS: Cardiac sarcoidosis
CT: Computed tomography
ECG: Electrocardiogram
FDG: 2-Deoxy-2-[^18^F]fluoro-d-glucose
FLT: 3′-Deoxy-3′-[^18^F]fluorothymidine
HRS: Heart Rhythm Society
ICC: Intraclass correlation coefficient
JMHW: Japanese Ministry of Health and Welfare
Kg: Kilogram
LV: Left ventricular
MBq: Megabecquerel
[^13^N]NH_3_: ^13^N-ammonia
NS: Neurosarcoidosis
PET: Positron emission tomography
^82^Rb: Rubidium-82
SUV: Standardized uptake value
