# Supplementary material for: FLT-PET for the assessment of systemic sarcoidosis including cardiac and CNS involvement: a prospective study with comparison to FDG-PET
Source: EJNMMI Res. 2020 Dec 10;10:154. doi: 10.1186/s13550-020-00742-x (PMC7728930; doi:10.1186/s13550-020-00742-x)
Supplement: Supplementary file 3 — Additional file 3. Table S3: Comparison of both readers' overall interpretation of the FLT- and FDG-PETs for neural involvement [FLT κ = 1.0 (95% CI 1.0–1.0), FDG κ = 1.0 (95% CI 1.0–1.0)]. [file 13550_2020_742_MOESM3_ESM.docx]

Supplementary Table 3. Comparison of both readers' overall interpretation of the FLT- and FDG-PETs for neural involvement (FLT κ = 1.0 (95% CI: 1.0 - 1.0), FDG κ = 1.0 (95% CI: 1.0 - 1.0))

| FLT-PET | | Reader 1 | |  |
| --- | --- | --- | --- | --- |
|  |  | Positive scan consistent with active NS | Negative scan | Total |
| Reader 2 | Positive scan consistent with active NS | 1 | 0 | 1 |
|  | Negative scan | 0 | 13 | 13 |
|  | Total | 1 | 13 | 14 |
| FDG-PET | | Reader 1 | |  |
|  |  | Positive scan consistent with active NS | Negative scan | Total |
| Reader 2 | Positive scan consistent with active NS | 1 | 0 | 1 |
|  | Negative scan | 0 | 13 | 13 |
|  | Total | 1 | 13 | 14 |

NS=neurosarcoidosis
